# Cremation

**Published:** 1880-10

**Authors:** Wm. Porter

**Affiliations:** St. Louis


### Selections.

---

CREMATION. By DR. WM. PORTER, of St. Louis.

There are few questions so generally misunderstood, so remunerative to the careful investigator, as is cremation. No custom or rite has a deeper hold upon our civilization than that of burial. The grave has become sacred through the sorrow of millions; religion has hallowed it; sentimental imagination has pictured "its quiet rest," "nor couldst thou wish couch more magnificent" than we are taught that is, around which our poets have twined the ivy and myrtle. Well might we hesitate to strip the "silent hall" of its drapery and reveal the sepulcher "full of dead men's bones." There is a duty, however, beyond the call of custom, sentiment or our preconceived ideas of tender regard for the dead.

The welfare of the living should guide us in disposing of the dead. This thought naturally leads us to contrast the practice of burial with the proposed one of cremation. The struggle between the two methods is mainly from a sanitary standpoint—other considerations are of minor importance.

A body is to be resolved into its original constituents. The water, carbonic acid gas and ammonia are to be separated from the more solid factors. This is the end to which all methods tend, whether the body be buried, burned, or left untouched upon the earth's surface. How can this be done safely, economically, and without violence to our feelings? We can not well be careless about the solution of this problem, for we have to deal not with one body alone, but with many thousands, and they are at our very door.

Take a single instance. The form of a dear one is placed in a coffin and buried from sight. From time to time the grave is visited, flowers are planted upon it, and deeply graven upon the marble is the sad story. We think of the silent sleeper, and in fancy gaze upon his upturned features. What is the reality? Down deep in the closely packed earth, so deep that nature's scavenger, the worm, refuses to prey—for worms do not "devour this mortal body" in our modern graves—shut off from the chemical effects of light and heat, a fearful process progresses. It is not enough to say we do not realize this, and think of the dead one as we last saw him. The change goes on whether we are sensible of it or not. The rain slowly percolates through the soil and accumulates, for most graves contain water. The festering ruin is macerated and the horrid infusion of death passes on into a water supply. The cemetery is beautifully situated upon a hillside, perhaps. Many cases of fever, "cause unknown," occur in the village below. The burying goes on, and we are soothed by the confident assertion that the earth is a deodorizer and disinfectant, no proof of harm has ever been given, there is no danger. Yes, but the proof has been given and there is danger.

Earth is a disinfectant only when thoroughly mixed with the decomposing substance. Even then it does not act freely unless the air has ready access to it. These conditions are wanting in our modern graves, and so long as that putrid mass is there, there is danger; do but touch it with your finger and if there be an abrasion you will probably die.

It is from ten to fifteen years ere complete disintegration of a human body, as ordinarily interred, is accomplished. A moderate estimate of the dead of St. Louis is 5,000 each year—50,000 in ten years. Think of 50,000 festering corpses within our city limits! No danger there? You can not trace disease to this source, say you? You can not always directly prove that defective sewage produces disease in a given case, but if common sense doesn't teach it, a sad experience may.

The Paris cemeteries are held responsible, by high scientific authority, for repeated epidemics of malignant sore throat and diarrhœa, if nothing worse, and yet sanitary science is in its infancy.

The strong arm of law in many of the older countries of Europe forbids human interment within a certain distance of dwellings, and cemeteries are removed by edict as a city grows. Strange is it that a government protects the health of its citizens, while physicians, whose province it is to investigate and guard against sources of disease, ignore the subject.

Happily this apathy is passing away. The merest tyro is not now ignorant of possible danger from inhumation, and as the sanitary physician brings new facts to light, increased opposition to burial as now practiced is developed.

What are some of these facts? Twenty years ago an epidemic of typhoid fever in Washington was traced to decomposing animal matter commingling with the water supply. Physicians began to think and to act. In 1875 the Massachusetts State Board of Health—one of the most reliable in the world—showed that typhoid fever is largely originated and diffused by emanation from bodies dead of that disease. Two years later the same high authority reached similar conclusions regarding diphtheria. Commenting upon this, the late Dr. LeMoyne says: "The inhumation of human bodies, dead from these infectious diseases, results in constantly loading the atmosphere and polluting the waters with not only the germs that arise from simple putrefaction, but also with the specific germs of the disease from which death originated."

Within my own knowledge is a case in point. A young man died suddenly from diphtheria and was buried in the village church-yard. At some little distance was a well from which the good church-goers drank freely each Sunday. Finally the water of the well became fœtid, for the supply was infiltrated by the horrible decomposition from this, the nearest grave. Was it not suggestive that twenty from that little congregation died from diphtheria while this impure well was in use? These people lived in mountain homes, in a pure atmosphere, and though many of these cases were isolated—far removed from others—yet in all the disease was alike virulent and deadly.

Those opposed to cremation tell us that if the laws of hygiene are properly understood and obeyed a graveyard may be so guarded as to be harmless. Possibly, but these laws are not understood and will never be fully obeyed; but even were it so what right have we to place a hidden quicksand in our neighbor's path and then wash our hands, because the danger has been fenced in by what we believe to be hygienic conditions? How difficult it is to impose such conditions even were they sufficient, or to legislate in the matter, is shown in Baker's laws relating to burial.

The growth of cities is constantly changing the site of our cemeteries. "Rest in the grave" and "undisturbed sleep" is an hypothesis, not a fact. You have seen it in our own city, the sacred inclosure is needed for city lots, a fashionable street is made to traverse "God's acre." Old coffins are thrust out to make room for a water main and the grave is replaced by a cellar, where the good wife stores her milk and potatoes. Now and then a bone may come to light but it is soon carted off after its fellows. The dead have not become unworthy in the lapse of years that they are thus disturbed; it is but a practical illustration of the principle already noted, that the depositing of the dead should be in the best interest of the living. This principle should apply as readily to the dead of yesterday as to the dead of years ago.

If we admit that inhumation is in any sense dangerous shall we avoid such danger by cremation? In this process the body is placed in a retort, not among the coals and ashes of the furnace as many suppose. In the Siemens' furnace, probably with some modification the best, super-heated air and hydro-carbon from an ordinary gas-retort are used. "The main retort is raised to a strong heat before the body is introduced, and after it is in place and the door closed, the amount of gas used for combustion is gradually diminished, as that coming from the body is sufficient." There is no foul vapor, and any germs of disease are destroyed, for it is a scientific fact that these germs cannot live beyond a certain temperature. During the process the body may be covered with asbestos, or cloth steeped in alum, which preserves the form till the cremation is complete. Think for a moment what a blessing such a practice would be in the plague-stricken cities of the South, or in the over-crowded ones of the old world.

Here, in place of the slow, and, as we believe, dangerous reduction of "earth to earth," we have the shorter and safer one of "ashes to ashes." You accomplish in an hour that which by burial requires a score of years. You have solved the problem, driven off the water, ammonia and carbonic-acid gas, destroyed the germs of disease, and have a more definite preservation of the solids of the body than is possible in any grave.

Probably the strongest opposition to cremation results from the horror of "being burned up," and a tender sentimentalism associated with our buried dead. This demands respect. Were the grave what it seems to be, were it within what it is upon the surface, this opposing ground would be well chosen. Remove a few feet of soil from the surface of our Bellefontaine, and note that change in the coffin since you last saw it. I well remember, when a boy, seeing our old sexton exhume a body burried for several years, that of a strong man, called away in the prime of life. The rotting coffin was slowly lifted from its damp bed, and the lid being broken we saw within a horrible mass of putrefaction. Matted hair and decomposing grave clothes but poorly covered the blackened skeleton as it lay in the once handsome casket, now reeking with the emanation of its loathsome contents. Yet this had been a beautiful grave; roses had bloomed upon it and the arbor-vitæ had whispered to it. There would be but little plea for the grave on the ground of sentiment could we see the changes there taking place; there would be few, if any, who would not choose that the body, after faithful service, should be purified by fire, rather than left to rot in such a grave.

Cremation has been advocated upon the score of economy. Our funerals entail great expense, and tombs are costly. I have seen the living pinched for their daily bread to pay the funeral expenses of the dead. In the crowded cities of Europe, where the cemeteries are necessarily at a distance, it is by a great sacrifice that the poor and sometimes the middle classes can provide for even the most unpretentious burial. The body of a London cockney is carried, at comparatively great expense, by his family out of the city which in his lifetime he was too poor to leave for a single day.

Granting, however, the objection, which is not by any means proven, that an equal amount of useless expenditure would be indulged in if cremation were adopted, as attaches to the present method, yet this remains: The vast acreage devoted to cemeteries and grave-yards represent sufficient value to provide crematories in every city and village in the land, and the proceeds of this land would be amply sufficient for the work of cremation wherever adopted. Further than this, the land would be productive—a matter of no little moment in some countries.

An objection to cremation has been raised on the ground that it is a heathen custom, opposed to the practice of the early Christian church. Will not our conscience be void of offense (in this respect, at least) toward the God who is the God of the living and not of the dead, if we dispose of the dead in the interest of the living? The great Founder of the church was not buried as we bury men, but was embalmed as, possibly, his ancestors had learned centuries before in Egypt. Many Christian martyrs have been cremated—not by their friends, it is true, but the process was none the less complete—and their ashes are held sacred. I know not if, in the Bible or among the teachings of holy men, there is one word to indicate one method of disposing of human remains as preferable to another.

It is true that a recent English writer says that the argument from religion is "dead against cremation, the few hints of the inspired writers all pointing to the earth as the final resting-place of the dead." We answer, inspired writers used similes in keeping with the customs of the age in which they lived. As well might we say that certain portions of the Bible taught that we should live in tents or tread out corn with oxen.

A further objection to cremation is that it would destroy all traces of criminal poisoning. True, but if cremation were instituted, there would be more careful scrutiny of dead bodies, and more official examinations. The detection of crime would be rather aided than hindered. Besides, we have no right to advocate a more or less general system of poisoning by inhumation that we may in rare cases discover one who has been the victim of poison. As recently shown by an honored member of this Society, such discoveries do not amount to much at best.

Public sentiment is practically opposed to cremation, and yet among our more thoughtful citizens there is a strong current in its favor. I have been surprised at the expression of this feeling—not alone as evidenced by your Society, gentlemen, but from many who are ready with you to practically indorse your action. This much in our own city. Abroad, in London, Vienna, Berlin, Padua, Paris, Leipsic and elsewhere, societies are now well established. The subject is receiving more thought and more favor than at any previous time. We are told that cremation can never be adopted. The London *Spectator*, of recent date, asserts that it has lost its force, never having made much progress. This is at variance with fact. Cremation is now being advocated principally upon sanitary grounds, and sanitary laws are only beginning to be understood. Much is yet to learn, but the little already known favors cremation. However, the *Spectator* sees enough of life in the cremation movement to desire a compromise between it and the usual method of burial. Wisely thought of. The charge of enthusiasm and fanaticism is now being cast upon the advocates of cremation from some who would be content that science should "stand like Joshua's moon at Ajalon." Most of the advance in every department of knowledge has been made by enthusiasts, and the charge of fanaticism is generally the strongest argument of him who brings it, worthy of its source, and worthless in this age. The position assumed by this Society is strong and right. You have placed the living before the dead, and though you may have to deal with many a dead man, so far as reason is concerned, yet the principles involved will live. It is not founded upon fanaticism, but deep-rooted in a strong sense of right and justice.

